# Molecular profiling of melanoma brain metastases compared to primary cutaneous melanoma and to extracranial metastases

**DOI:** 10.18632/oncotarget.27686

**Published:** 2020-08-18

**Authors:** Gino K. In, Kelsey Poorman, Michelle Saul, Steven O’Day, Jeffrey M. Farma, Anthony J. Olszanski, Michael S. Gordon, Jacob S. Thomas, Burton Eisenberg, Lawrence Flaherty, Amy Weise, Steven Daveluy, Geoffrey Gibney, Michael B. Atkins, Ari Vanderwalde

**Affiliations:** ^1^USC Norris Comprehensive Cancer Center, Los Angeles, CA, USA; ^2^Caris Life Sciences, Phoenix, AZ, USA; ^3^John Wayne Cancer Institute, Santa Monica, CA, USA; ^4^Fox Chase Cancer Center, Philadelphia, PA, USA; ^5^HonorHealth Medical Group, Scottsdale, AZ, USA; ^6^Hoag Family Cancer Institute, Newport Beach, CA, USA; ^7^Karmanos Cancer Institute, Detroit, MI, USA; ^8^Georgetown Lombardi Comprehensive Cancer Center, Washington, DC, USA; ^9^University of Tennessee Health Science Center, West Cancer Center, Germantown, TN, USA

**Keywords:** melanoma, brain metastases, BRAF, PD-L1, TMB

## Abstract

Background: Brain metastases are a significant cause of mortality and morbidity for patients with melanoma. We hypothesize that the development of brain metastases may be explained by molecular heterogeneity between primary cutaneous melanoma (PCM) or extracranial (ECM) and brain (MBM) melanoma metastases.

Materials and Methods: We compared next-generation sequencing, tumor mutational burden (TMB), and immunohistochemical staining for PD-L1 expression, among 132 MBM, 745 PCM, and 1190 ECM.

Results: The most common genetic alterations among MBM included: BRAF (52.4%), NRAS (26.6%), CDKN2A (23.3%), NF1 (18.9%), TP53 (18%), ARID2 (13.8%), SETD2 (11.9%), and PBRM1 (7.5%). Four genes were found with higher frequency among MBM compared to PCM or ECM: BRAF (52.4% v 40.4% v 40.9%), SETD2 (11.9% v 1.9% v 3.9%), PBRM1 (7.5% v 1.6% v 2.6%), and DICER1 (4.4% v 0.6% v 0.4%). MBM showed higher TMB (*p* = .04) and higher PD-L1 expression (*p* = .002), compared to PCM. PD-L1 expression was slightly higher among MBM compared to ECM (*p* = .042), but there was no difference between TMB (*p* = .21).

Conclusions: Our findings suggest a unique molecular profile for MBM, including higher rates of BRAF mutations, higher TMB and higher PD-L1 expression, and also implicate chromatin remodeling in the pathogenesis of MBM.

## INTRODUCTION

The incidence of melanoma continues to rise, with nearly 100,000 new cases occurring each year in the United States alone [1]. Among patients with advanced melanoma, approximately 50% develop brain metastases, resulting in significant morbidity and mortality [2–5]. Localized therapies, including surgery and radiation, have historically resulted in overall survival of 4-6 months for patients with melanoma brain metastases (MBM) [3, 5–7].

Recent clinical trials demonstrate the efficacy of systemic therapy in the treatment of MBM. Two studies, the Anti-PD1 Brain Collaboration (ABC) and CheckMate 204, treated patients using the combination of two checkpoint inhibitors (CPI), anti-CTLA-4 and anti-PD-1; this resulted in higher response rates compared to either CPI alone, and with intracranial efficacy similar to that seen in extracranial metastases [8, 9]. Unfortunately, there is no standardized biomarker to identify which MBM patients will best respond to CPI. While the ABC study demonstrated improved outcomes for patients with PD-L1 expression greater than 1%, CheckMate 204 found no difference between patients with PD-L1 expression greater than or less than 5%.

In addition to being highly immunogenic, another defining characteristic of melanoma is constitutive activation of the MAPK pathway, via BRAF V600 mutations. COMBI-MB study was the first clinical trial to demonstrate the efficacy of targeted BRAF+MEK inhibition for patients with BRAF mutated MBM; again, intra-cranial response rates were similar to that seen when treating extracranial metastases (ECM) [10]. However, responses to BRAF+MEK inhibition were less durable (median progression free survival 5.6 months) than responses in ECM, and mechanisms of resistance were not identified in this study.

To date, primary melanomas (PCM) and ECM have been extensively studied; in contrast, the biology of MBM remains poorly understood, largely due to lack of available tissue. Increasing evidence supports the notion that distinct tumor clones evolve throughout tumor progression, e.g., when comparing primary against metastatic tumors [11–14]. Such tumor heterogeneity may drive both development of metastatic disease, as well as resistance to cancer therapy. Brastianos and colleagues performed whole-exome sequencing among 86 matched primary tumors and brain metastases (including melanoma and other tumor types), and in doing so, identified unique genetic alterations between the matched pairs in more than 50% of cases [11]. In MBM specifically, upregulation of the PI3K-AKT pathway has been consistently identified, suggesting its role in the pathogenesis of these tumors [15–17]. Furthermore, extensive research has defined the unique features of the brain as an anatomic site for metastatic seeding, encompassing: i) the blood brain barrier [18–20], ii) the diversity of neuronal cell types involved (e.g. astrocytes, microglia) [21–24], and other factors modulating iii) tumor cell transmigration and adhesion [18, 19, 25], iv) extracellular matrix degradation [23, 26–30], and v) angiogenesis [27, 29, 31–33]. Improved understanding of the complex pathogenesis of MBM is needed to help improve clinical therapeutic approaches.

To address these gaps, we performed a cross-sectional analysis of melanoma samples with comprehensive molecular profiling available via the Caris Life Sciences database, to examine for differences between MBM, when compared to PCM and other ECM.

## RESULTS

### Patient demographics

**Figure 1:**
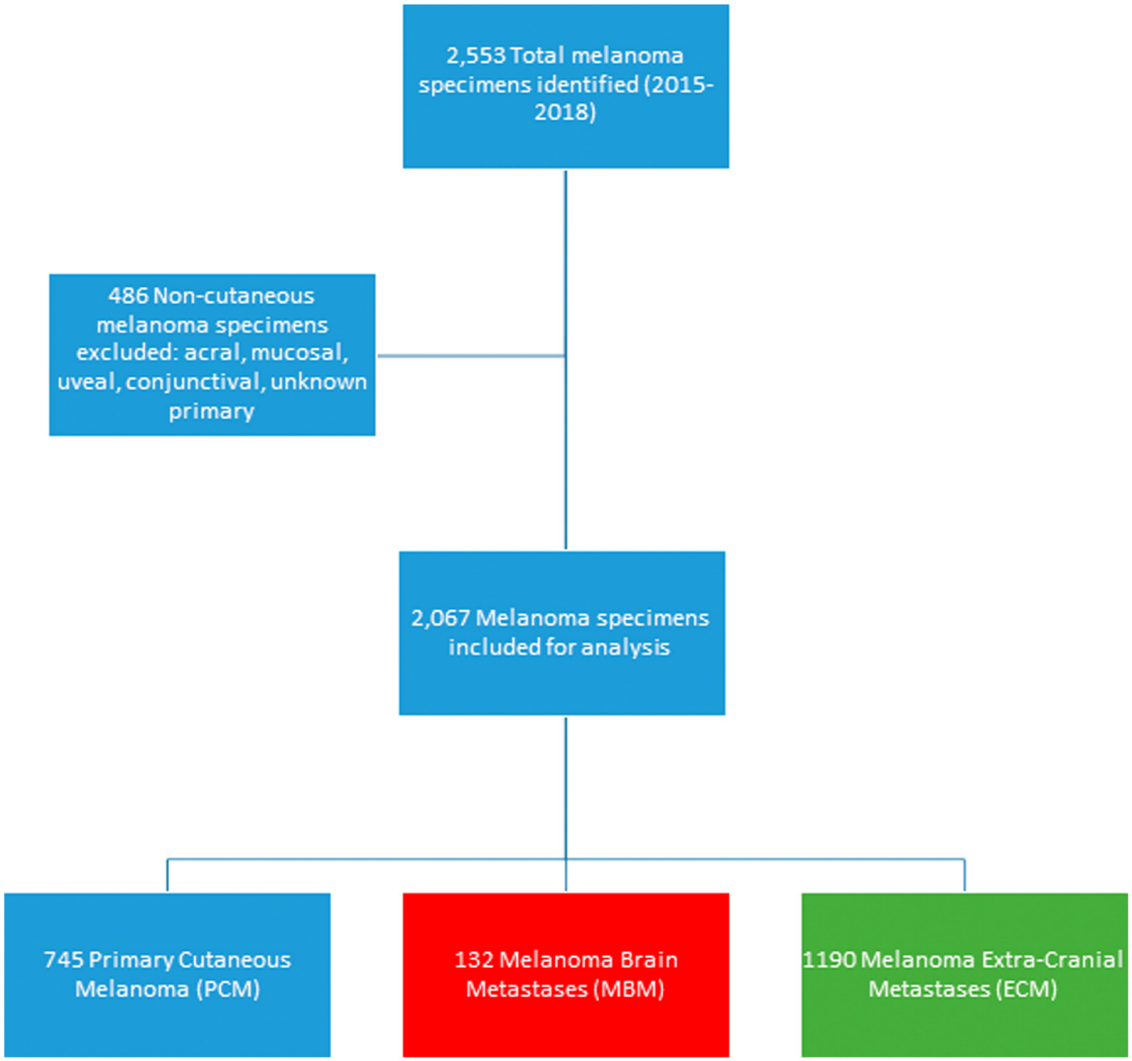
Consort diagram of melanoma specimens included in the study.

A total of 2,067 cutaneous melanoma samples were included in this analysis: 132 MBM, 745 PCM and 1190 ECM (Figure 1). Among the 132 MBM samples, 48.5% originated from the supratentorial region of the brain, while 4.6% derived from infratentorial regions, 11.4% from brain stem, and 35.6% of samples did not have information specific to location.

### Mutational profiling via next-generation sequencing

**Figure 2:**
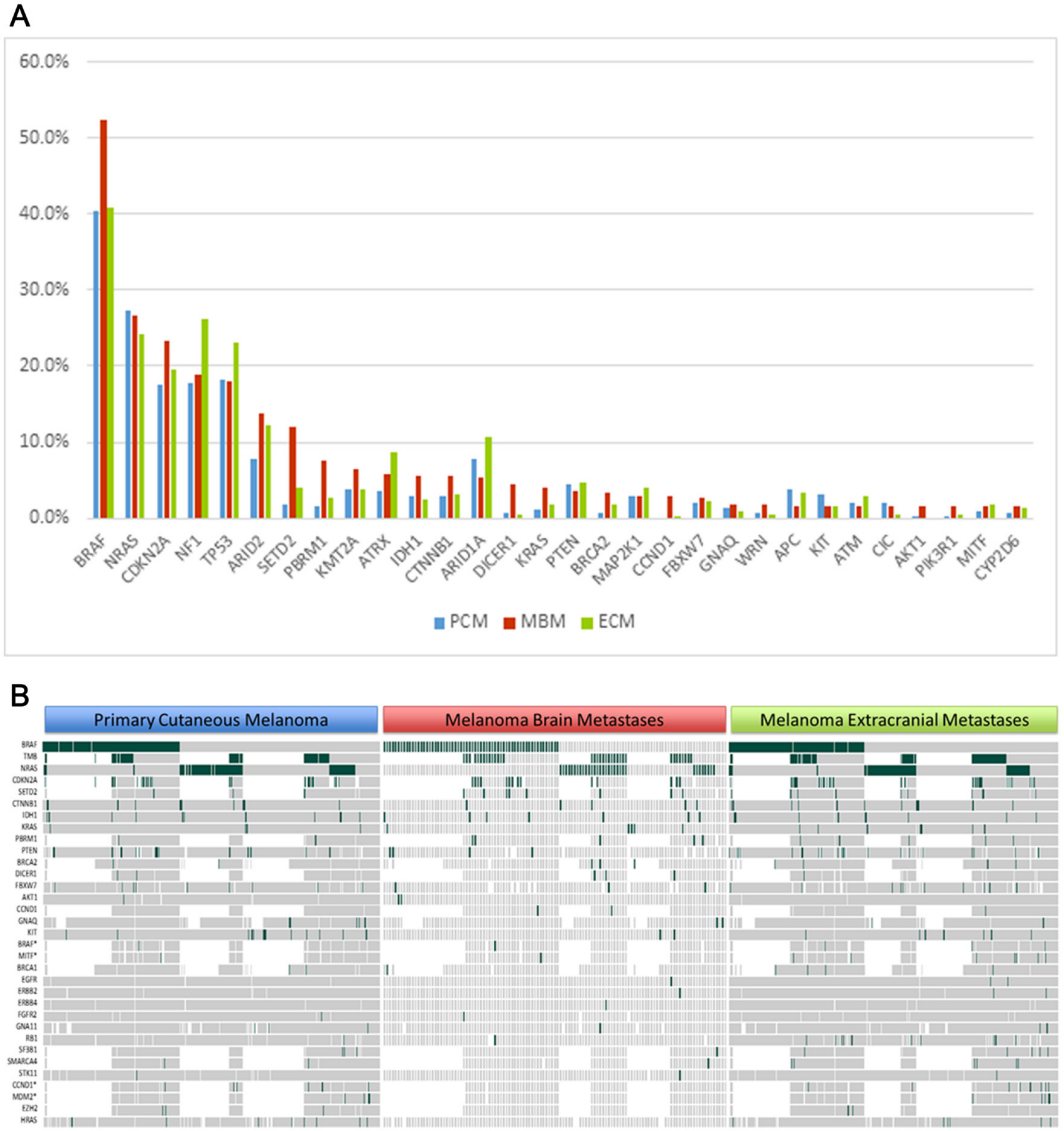
Comparison of gene alterations between primary cutaneous melanoma (PCM), melanoma brain metastases (MBM), and extracranial metastases (ECM). (**A**) Genes are listed in descending order of the 30 most frequent alterations identified in MBM. (**B**) Heatmap visualization of genomic alterations found across anatomic sites. Copy number variants denoted by asterisk (^*^).

The most common mutations occurring among PCM and ECM samples, respectively, were BRAF (40.4%, 40.9%), NRAS (27.3%, 24.2%), TP53 (18.2%, 23.0%), NF1 (17.9%, 26.3%) and CDKN2A (17.6%, 19.6%), consistent with prior studies (Figure 2A). Among MBM samples as well, the most frequently altered genes were: BRAF (52.4%), NRAS (26.6%), CDKN2A (23.3%), NF1 (18.9%), and TP53 (18.0%). Following these top 5 genes, the most common altered genes, occurring in at least 5% or more of all MBM samples, were the following: ARID2 (13.8%), SETD2 (11.9%), PBRM1 (7.5%), KMT2A (6.6%), ATRX (5.9%), IDH1 (5.6%), CTNNB1 (5.6%), and ARID1A (5.3%).

However, when analyzing MBM against the PCM cohort, the following genes were more frequently altered among MBM: SETD2 (11.9% v 1.9%, *p* = .0008), BRAF (52.4% v 40.4%, *p* = .017), PBRM1 (7.5% v 1.6%, *p* = .018), KRAS (4.0% v 1.0%, *p* = .026), CCND1 (2.9% v 0%, *p* = .031), and DICER1 (4.4% v 0.6%, *p* = 0.04) (Figure 2B). When analyzing MBM against ECM, higher rates of mutations were observed among: SETD2 (11.9% v 3.9%, *p* = .009), DICER1 (4.4% v 0.4%, *p* = .011), AKT1 (1.6% v 0%, *p* = .011), BRAF (52.4% v 40.9%, *p* = .019), and PBRM1 (7.5% v 2.6%, *p* = .049).

### Tumor mutational burden and PD-L1

**Figure 3:**
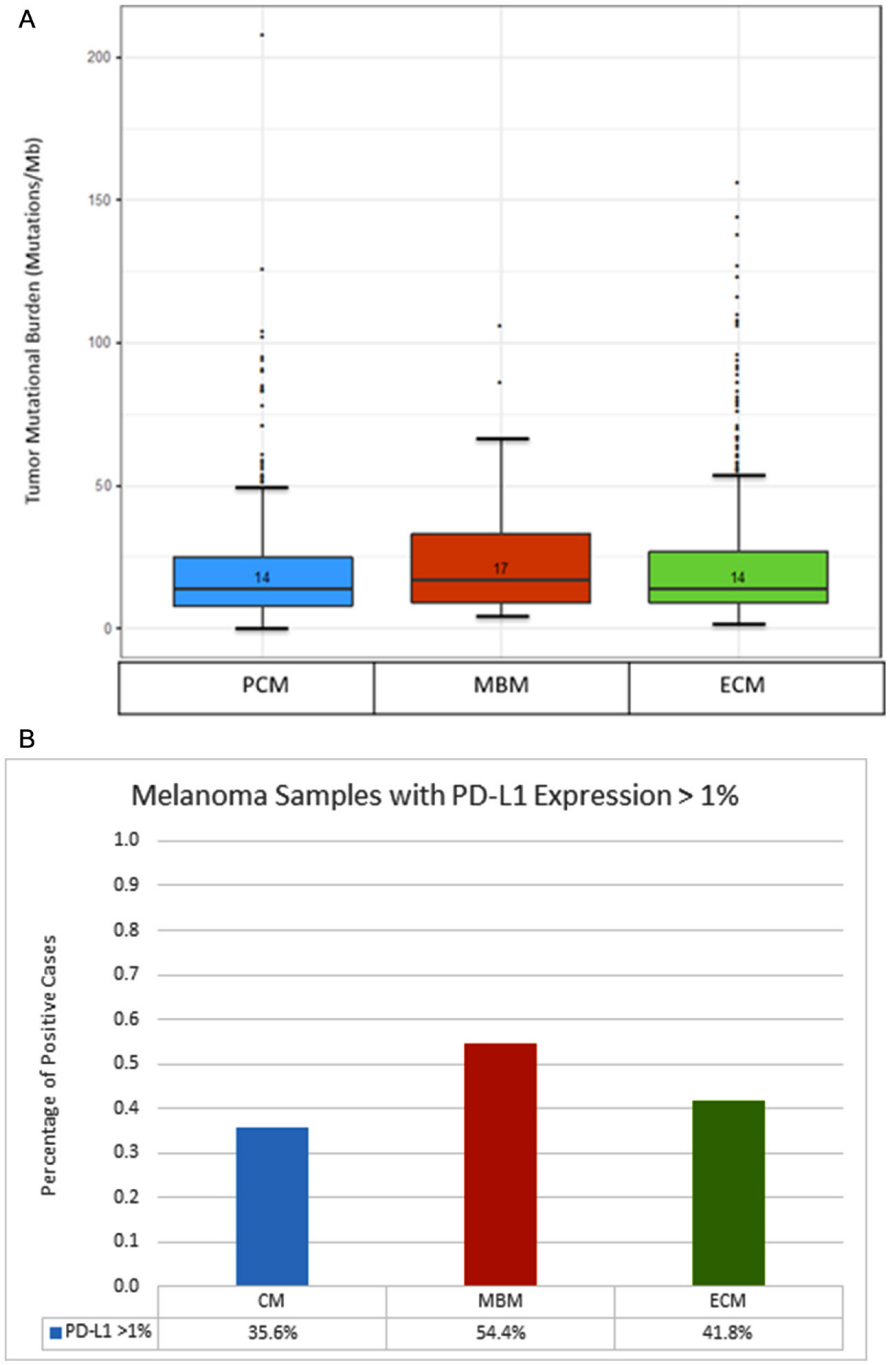
Comparison of tumor mutational burden and PD-L1 expression between primary cutaneous melanoma (PCM), melanoma brain metastases (MBM) and extracranial metastases (ECM). (**A**) Box plot of tumor mutational burden (TMB) across distinct anatomic sites. Median TMB is listed above the bar. (**B**) Frequency of Tumor Samples with PD-L1 Expression Greater than 1%, Across Anatomic Sites.

The median TMB for MBM was 17 mutations/Mb, while median TMB for PCM was 14 mutations/Mb, and median TMB for ECM was 14 mutations/Mb (Figure 3A). By Mann Whitney testing analysis, TMB was higher for MBM compared to PCM (*p* = .04), but there was no statistical difference when comparing MBM to ECM (*p* = .21).

IHC analysis revealed higher PD-L1 expression among MBM, compared to PCM, using a 1% cutoff (54.4% v 35.6%, *p* = .002) (Figure 3B). There was also a potential difference in PD-L1 expression when comparing MBM against ECM (54.4% v 41.8%, *p* = .042), and comparing ECM against PCM (41.8% v 35.6%, *p* = .048).

### Signaling pathway analysis by anatomic site and molecular subgroup

Pathway analysis of melanoma samples by anatomic site revealed higher rates of mutations affecting the MAPK pathway among MBM, compared to PCM (87.9% v 77.8%, *p* = .015) and compared to ECM (87.9% v 77.5%, *p* = .011). The SWI/SNF pathway was also enriched with more alterations when comparing MBM to PCM (22.1% v 11.6%, *p* = .036), but not so when comparing MBM to ECM (22.1% v 17.8%, *p* = .49). (Supplementary Table 1).

Given the high frequency of alterations among the methylation, histone modification and SWI/SNF pathways collectively, we performed an overall grouped analysis for all pathways that impact chromatin modification. This grouped chromatin pathway analysis showed enrichment for more genetic alterations among MBM compared to PCM (23.4% v 12.3%, *p* = .002); however, there was only a trend towards significance when comparing MBM to ECM (23.4% v 16.2%, *p* = .06).

We then analyzed the 7 specific pathways among MBM samples stratified by TCGA molecular subgroups. Amongst BRAF mutated MBM, there was a higher frequency of alterations among the PIK3-AKT pathway, compared to BRAF wild type (20.0% v 5.1%, *p* = .027). Amongst the NF1 mutated subgroup, we noted higher rates of alterations among the SWI/SNF pathway, compared to NF1 wild type (60.0% v 11.6%, *p* = .003). No significant pathway differences were noted for the NRAS or triple wild type subgroups.

### Mutational analysis of matched melanoma specimens

**Figure 4:**
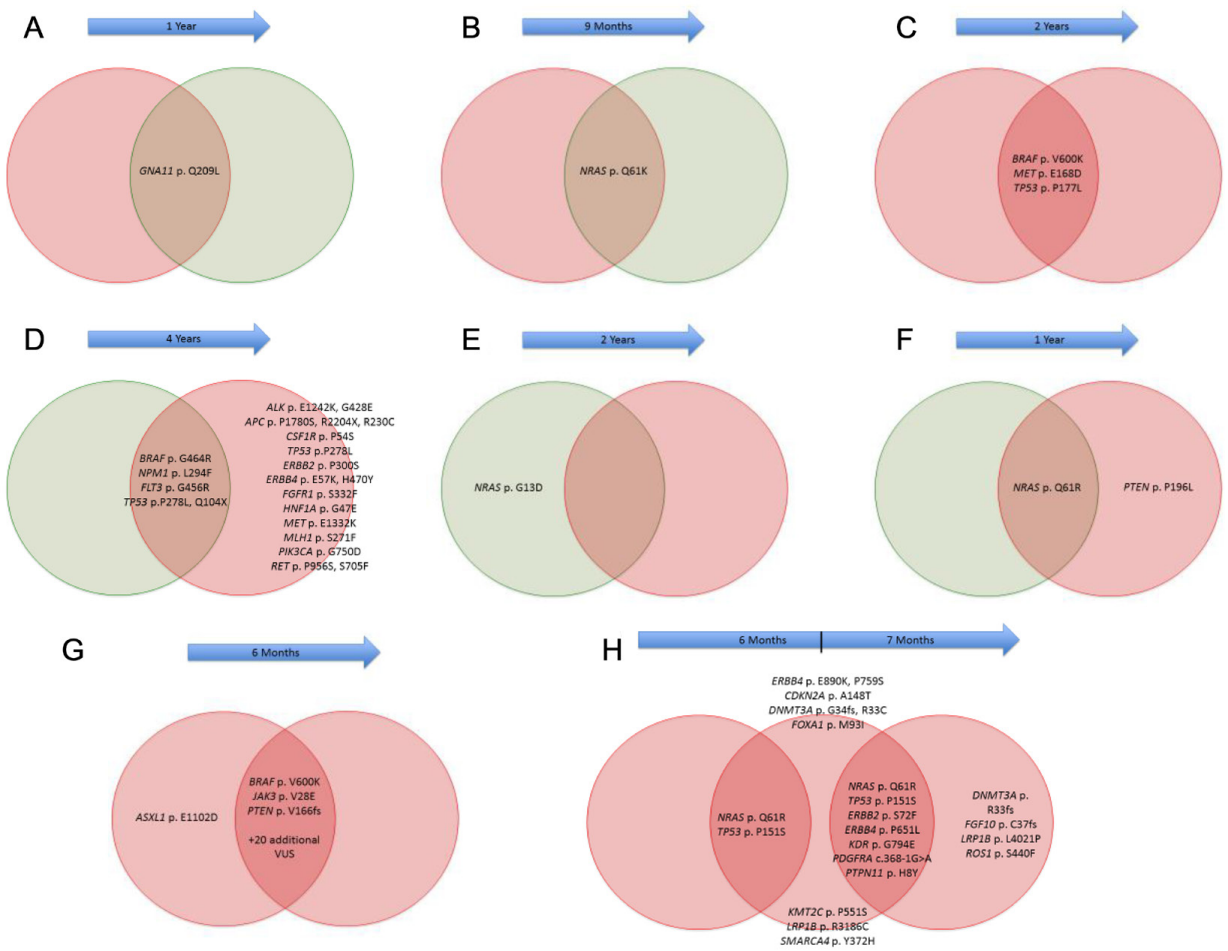
Comparison of gene alterations between matched melanoma samples. Melanoma brain metastases (MBM) denoted in red, while extracranial metastases (ECM) denoted in green. Blue arrows indicate time between development of each metastasis.

Matched melanoma specimens were available for 8 patients with MBM within our cohort (Figure 4). Among these, 1 patient had a corresponding primary cutaneous melanoma tumor, while 3 had matched lymph node metastases, and 2 had matched lung metastases. There were 3 patients with multiple matched MBM. Among patients with matched samples, at least 5 demonstrated unique genetic alterations between anatomic sites. The first patient was found to have BRAF, FLT3, NPM1 and TP53 mutations within a lung metastasis, while the matched brain tumor had these same mutations, as well as 12 other mutations. A second patient harbored an NRAS mutation in a lymph node metastasis, while the matching brain metastasis was NRAS wild type. The third patient had an NRAS mutation detected in both lymph node and brain metastases; however, the lymph node was wild type PTEN while the matched brain metastasis was PTEN mutated. The fourth patient had a BRAF mutation found in 2 separate MBM, as well as the same mutations affecting another 22 genes; however, a single gene, ASXL1, was mutated in 1 brain metastasis, but wild type in the other brain metastasis. In a fifth patient with 3 different MBM, NRAS and TP53 mutations were found in all 3 tumors, while 2 tumors harbored mutations in 5 other genes (ERBB2, ERBB4, KDR, PDGFRA, PTPN11), all of which were wild type in the 1st tumor; in addition, 6 other genes were also found to have distinct mutations between these other 2 tumors. No differences were noted for the remaining patients.

### DISCUSSION

Brain metastases lead to significant mortality and morbidity for patients with advanced melanoma, and improved understanding of the biology of MBM remains a major unmet need. Prior investigations of MBM have been restricted by low sample size numbers or limited by the scope of analyses performed. Our purpose was to leverage a large multi-center, clinical repository of melanoma tumors, for which multi-platform molecular testing, including PD-L1, TMB, and NGS were simultaneously available. We must note several important limitations to this work. First, the Caris Life Sciences tissue repository consists of tumor samples for which molecular profiling was conducted to aid in clinical decision making; as such there may be over-representation of tumors for which no standard treatments are available. Second, our analysis does not include clinical outcomes data to correlate with the molecular findings reported. Third, our NGS analysis was limited to a pre-specified panel of cancer-related genes, and reports findings at the genomic level alone; we were unable to corroborate our findings at the RNA or protein level. Nevertheless, our work presents one of the largest analyses of MBM to date, and should be considered in this context.

Prior studies have demonstrated upregulation of the MAPK pathway among MBM. While our analysis found a higher rate of BRAF mutations among MBM relative to PCM and ECM, we did not find a significant difference in the frequency of NRAS mutations; this contrasts with the findings of Colombino et al., who found that the frequency of *both* BRAF and NRAS mutations was highest among MBM, compared to PCM or other ECM [34]. Again, we note the selection bias inherent to this database, which may have skewed the distribution of driver mutations among the samples presented. Nevertheless, our results showed alterations among genes grouped to the MAPK pathway, overall, in nearly 80% of MBM samples. In addition, when stratified by molecular subgroups, we identified recurrent alterations of the PI3K/AKT pathway among the BRAF subgroup. This is consistent with prior studies that describe activation of the PI3K/AKT pathway in the pathogenesis of MBM, and also implicate this pathway as a possible resistance mechanism to BRAF inhibition [15–17]. At least one study, by Bucheit et al., found that PTEN loss within BRAF V600 mutated melanomas was associated with significantly shorter time to development of MBM [15]. In the COMBI-MB study, responses to BRAF+MEK inhibition were less durable among MBM compared to ECM (median duration of response 6.5 v 10.2 months). Patients from COMBI-MB also had shorter progression free survival (median 5.6 months), than other BRAF+MEK studies that excluded MBM (median 9.3–14.9 months). Our findings suggest that the limited efficacy of BRAF+MEK inhibition in MBM may, at least in part, be due to molecular differences (i.e. PI3K/AKT activation) that exist between these anatomic sites.

Unlike lung cancer and other tumor types, the utility of PD-L1 as a predictive biomarker in patients with melanoma (and MBM) remains unclear. While high PD-L1 expression is felt to indicate a highly inflamed tumor that is more likely to respond to CPI, lack of PD-L1 expression does not preclude a response to therapy. In our study, we found higher PD-L1 expression among MBM compared to PCM when using a cutoff of 1%; we also noticed that PD-L1 expression may be higher for MBM compared to ECM, as well as for ECM compared to PCM. This contrasts with a study by Kluger et al., who reported a trend towards lower PD-L1 expression among MBM compared to other ECM, although this did not meet statistical significance [35]. In a separate study, Fischer et al. found no difference in PD-L1 expression between MBM and ECM, although the authors did report up to 40% discordance between a small subset of matched samples [36]. It should be noted that both of these studies employed a different PD-L1 antibody and scoring system from ours. Furthermore, the study by Kluger and colleagues separated ECM by anatomic sites (skin, soft tissue, lymph node, visceral, etc). Another difference is that both of the aforementioned studies noted that lower PD-L1 expression was associated with decreased tumor infiltrating lymphocytes (TILs) among MBM. TILs may be present independently of PD-L1 expression, and thus are also being explored as a potential biomarker for checkpoint blockade. Unfortunately, our platform did not allow us to comment on the presence of TILs, which may also explain differing response rates to CPI between MBM and ECM.

In addition to PD-L1, we explored TMB among MBM as another potential biomarker for response to treatment with CPI. Interestingly, we noted higher TMB among MBM compared to PCM, although TMB was not significantly higher for MBM when compared to ECM. To our knowledge, this is the first study to specifically describe TMB among MBM. While prior investigations show that higher TMB may be predictive of response to CPI in melanoma and other tumors, these studies differ in regards to the platform and thresholds used for assessing TMB, and also do not distinguish TMB across anatomic sites [37–45]. It should be noted that in our cohort, we did find high TMB levels for the upper limit range across all the compared anatomic subgroups. We did not appreciate a difference in TMB among the small subset of matched samples within our cohort; this is consistent with findings reported by Fischer et al., who performed whole-exome sequencing to measure overall rates of nonsynonymous mutations among matched samples, but found no difference between matched MBM and ECM [36]. Of note, the authors did not find a correlation between mutation rate and the presence of TILs within MBM [36], perhaps suggesting that TMB alone may not be predictive of immune response for brain metastases. Another finding, also concordant with prior work, was that patients within the NF1 subgroup had the highest TMB, while patients with BRAF and NRAS mutations had lower TMB [39, 46–48].

Our analysis demonstrated a number of genetic alterations that have a role in epigenetic modulation, which had not previously been described in the context of MBM. We noted higher rates of alterations in SETD2, which is involved in histone modification, and PBRM1, which encodes the BAF 180 subunit of the PBAF SWI/SNF chromatin remodeling complex [49, 50]. Furthermore, we detected recurrent alterations among the SWI/SNF pathway, as well as the overall grouping of all 3 chromatin modulating pathways (histone modification, methylation, SWI/SNF) in MBM. In an NGS study using a panel of 275 cancer genes to study 38 melanoma samples (13 PCM, 25 metastatic samples), Lee and colleagues found that 22.3% of all mutations occurred in genes affecting epigenetic regulating pathways, while at least 1 mutation affecting epigenetic regulation was present in 92% of samples [51]. Of interest, we detected recurrent alterations among the SWI/SNF pathway for the NF1 molecular subgroup; this coincides with prior studies showing that NF1 loss may occur concurrently with ARID1A mutations [46, 52]. Again, we emphasize that these genetic alterations should be followed by transcriptomic and proteomic analyses, to understand their functional role in MBM, and examine whether chromatin modifying therapies (HDAC, DNMT, and EZH2 inhibitors) may have any potential therapeutic application.

Regrettably, we do not have clinical outcomes to correlate with the findings of our investigations. Thus, we are unable to delineate whether these MBM developed *de novo*, or as relapsed/refractory MBM following therapeutic intervention, nor can we comment on other clinical factors which may impact outcomes in metastatic melanoma. Another limitation of our study is the relative lack of matched samples. Although we did have a large cohort of MBM specimens, only a small minority were available as matched samples from the same patient, and thus we cannot comment on the possibility of intra-patient heterogeneity. Future studies should explore not only the molecular heterogeneity of MBM, but also other ECM, as there are likely further differences reflected between various anatomic visceral sites (e.g. liver compared to lung), as well as nodal and cutaneous metastases.

In conclusion, our analysis of a large cohort of MBM using multiplex testing demonstrated that two salient features of melanoma, including: a) upregulation of the MAPK pathway, and b) its tumor immunogenicity, appear consistent among MBM, but may be modulated by other molecular factors not found among PCM and ECM. We also describe the presence of multiple genetic alterations associated with chromatin remodeling among MBM, which may suggest a novel pathway to target. Overall our findings indicate that molecular heterogeneity exists between tumors/metastases at different anatomic sites. Further investigation is needed to validate these findings and elucidate their clinical applicability.

### MATERIALS AND METHODS

A total of 2,553 melanoma specimens submitted during routine clinical care were evaluated by comprehensive profiling at Caris Life Sciences (Phoenix, AZ) between January 2015 and October 2018. All specimens were grouped by primary tumor site and specimen site, as provided in tissue requisition requests. Tumor histology and diagnoses were confirmed centrally by board-certified pathologists based off formalin-fixed, paraffin-embedded (FFPE) sections. Acral, mucosal, conjunctival and uveal melanoma subtypes were excluded, as were melanoma of unknown primary and any melanomas without clear documentation of cutaneous origin. Cutaneous melanoma samples were then divided into 3 groups: PCM, ECM, and MBM. ECM included all non-CNS metastases, including visceral, skin/cutaneous, and nodal metastases. PCM were distinguished from skin/cutaneous metastases based on pathology reports and clinical documentation by the treating physician.

### Next-generation sequencing

Next-generation sequencing (NGS) was performed on genomic DNA isolated from FFPE tumor samples using the NextSeq (592-genes)/MiSeq platform (45-gene) to evaluate for DNA aberrations (Illumina NextSeq; Illumina, San Diego, CA). All variants were detected with greater than 99% confidence based on allele frequency and amplicon coverage, with an average sequencing depth of coverage greater than 500 and an analytic sensitivity of 5%. Copy number variants were generated only for cases profiled using the 592-gene panel. All molecular techniques met Clinical Laboratory Improvement Amendments/College of American Pathology standards.

### Tumor mutational burden

Tumor mutational burden (TMB) was calculated using methodology as previously described by Vanderwalde et al. and others [53, 54]. To summarize, the TMB was estimated from 592 genes (1.4 megabases [MB] sequenced per tumor) by counting all non-synonymous missense mutations found per tumor that had not been previously described as germline alterations. TMB was reported as a continuous variable.

### Immunohistochemistry

Immunohistochemistry (IHC) was used to assess protein expression; all IHC was performed on FFPE sections of glass slides. Positive and negative controls were included to ensure staining efficacy and consistency across batches. PD-L1 testing was performed using the SP142 (Ventana, Tucson, AZ) anti-PD-L1 clone as measured on tumor cells. PD-L1 positivity was evaluated using a cutoff of 1+ staining intensity on ≥ 1% of tumor cells.

### Signaling pathway analysis

We next explored for differences in genetic alterations grouped by signaling pathways, as compared between both anatomic sites (PCM, ECM, MBM), and TCGA molecular subtypes (BRAF, NRAS, NF1, triple wild type). Commonly altered tumor suppressor genes and oncogenes were assigned to 7 potential signaling pathways of interest, as defined by the Gene Ontology (GO) Consortium, and the Kyoto Encyclopedia of Genes and Genomes (KEGG). A pathway was considered altered if at least one of the assigned genes or oncogenes in the pathway contained a genetic alteration. The pathways were then stratified according to anatomic site, and by molecular subtype (among MBM only).

### Statistical analysis

Standard descriptive statistics were used for this retrospective analysis. For dichotomous outcomes, Fisher’s exact test or Pearson’s Chi-square test were performed. For continuous outcomes, Mann-Whitney *U* tests were conducted. A false-discovery rate adjustment was applied to *p*-values using the Benjamini-Hochberg method. Adjusted *p*-values < 0.05 were considered statistically significant. All analyses were conducted using R (version 3.5.0).

## SUPPLEMENTARY MATERIALS



## Abbreviations

ARID1A: AT-rich interaction domain 1A
ARID2: AT-rich interaction domain 2
ASXL1: ASXL transcriptional regulator 1
ATRX: ATP-dependent helicase ATRX
BRAF: v-Raf murine sarcoma viral oncogene homolog B1
CCND1: cyclin D1
CDKN2A: cyclin-dependent kinase inhibitor 2A
CTNNB1: catenin beta 1
DICER1: dicer 1, ribonuclease III
DNMT: DNA methyltransferase
ERBB2: erb-b2 receptor tyrosine kinase 2
ERBB4: erb-b2 receptor tyrosine kinase 4
EZH2: enhancer of zeste homolog 2
FLT3: fms-related receptor tyrosine kinase 3
HDAC: histone deacetylase
IDH1: isocitrate dehydrogenase 1
KDR: kinase insert domain receptor
KMT2A: lysine methyltransferase 2A
KRAS: Kirsten rat sarcoma viral oncogene homolog
MAPK: mitogen activated protein kinase
MEK: mitogen activated protein kinase kinase
NRAS: neuroblastoma RAS viral oncogene homolog
NF1: neurofibromatosis 1
NPM1: nucleophosmin 1
PI3K-AKT: phosphoinositide 3-kinase – protein kinase B
PBRM1: polybromo 1
PD-L1: programmed death-ligand 1
PDGFRA: platelet-derived growth factor receptor alpha
PTPN11: protein tyrosine phosphatase, non-receptor type 11
TMB: tumor mutational burden
TP53: tumor protein p53
SETD2: SET domain containing 2, histone lysine methyltransferase
SWI/SNF: SWItch/Sucrose Non-Fermentable
