# Supplementary material for: Molecular profiling of melanoma brain metastases compared to primary cutaneous melanoma and to extracranial metastases
Source: Oncotarget. 2020 Aug 18;11(33):3118–28. doi: 10.18632/oncotarget.27686 (PMC7443369; doi:10.18632/oncotarget.27686)
Supplement: Supplementary file 1 [file oncotarget-11-3118-s001.pdf]

Molecular profiling of melanoma brain metastases compared to primary cutaneous melanoma and to extracranial metastases

SUPPLEMENTARY MATERIALS

Supplementary Table 1: Comparison of altered pathways between primary cutaneous melanoma (PCM), melanoma brain metastases (MBM) and extracranial metastases (ECM)

(A) Comparison of altered pathways between PCM and MBM

Pathway	Representative altered genes	PCM with alteration of at least one gene, %	MBM with alteration of at least one gene, %	*p*-value
MAPK Signaling	*BRAF, NRAS, NF1, MAP2K1/2, ERK1/2*	77.8 (528/679)	87.9 (109/124)	.015
SWI/SNF	*ARID1A, ARID2, SMARCA4, PBRM1*	11.6 (37/319)	22.1 (15/68)	.036
Methylation	*TET2, IDH1/2*	2.8 (19/676)	5.6 (7/124)	.103
Histone Modification	*SETD2, KMT2A/C/D, EZH2, CREBBP*	10.1 (32/318)	17.6 (12/68)	.115
PI3K/AKT	*PIK3CA, PTEN, AKT1, MTOR, TSC1/2*	8.9 (62/699)	12.7 (16/126)	.235
Cell Cycle	*CKDN2A, CCND1, RB1, CDK4/6*	9.1 (61/668)	12.9 (16/124)	.256
DNA Damage	*TP53, MDM2, ATM, CHEK1/2*	20.6 (139/676)	19.4 (24/124)	.853
Chromatin^	NA	12.3 (83/676)	23.4 (29/124)	.002*

(B) Comparison of altered pathways between ECM and MBM

Pathway	Representative altered genes	ECM with alteration of at least one gene, %	MBM with alteration of at least one gene, %	*p*-value
MAPK Signaling	*BRAF, NRAS, NF1, MAP2K1/2, ERK1/2*	77.5 (821/1059)	87.9 (109/124)	.011
Methylation	*ARID1A, ARID2, SMARCA4, PBRM1*	2.5 (27/1059)	5.6 (7/124)	.079
DNA Damage	*TET2, IDH1/2*	26.8 (283/1056)	19.4 (24/124)	.093
Histone Modification	*SETD2, KMT2A/C/D, EZH2, CREBBP*	11.4 (65/572)	17.6 (12/68)	.191
SWI/SNF	*PIK3CA, PTEN, AKT1, MTOR, TSC1/2*	17.8 (102/572)	22.1 (15/68)	.492
PI3K/AKT	*CKDN2A, CCND1, RB1, CDK4/6*	11.3 (125/1105)	12.7 (16/126)	.753
Cell Cycle	*TP53, MDM2, ATM, CHEK1/2*	14.0 (147/1048)	12.9 (16/124)	.838
Chromatin^	NA	16.2 (172/1059)	23.4 (29/124)	.060

Signaling pathways listed in order of *p*-value significance. ^Chromatin categorization includes SWI/SNF, Methylation, and Histone pathways grouped together. *Asterisk indicates *q*-value < 0.05.
